# Quantifying the Cost of *Shigella* Diarrhea in the Enterics for Global Health (EFGH) *Shigella* Surveillance Study

**DOI:** 10.1093/ofid/ofad575

**Published:** 2024-03-25

**Authors:** Chloe Morozoff, Naveed Ahmed, Jobiba Chinkhumba, Md Taufiqul Islam, Abdoulie F Jallow, Billy Ogwel, Loyda Fiorella Zegarra Paredes, Doh Sanogo, Hannah E Atlas, Henry Badji, Naor Bar-Zeev, Bakary Conteh, Mario Güimack Fajardo, Erika Feutz, Fadima C Haidara, Mehrab Karim, Adama Mamby Keita, Youssouf Keita, Farhana Khanam, Margaret N Kosek, Karen L Kotloff, Rebecca Maguire, Ishmail S Mbutuka, Maureen Ndalama, John Benjamin Ochieng, Collins Okello, Richard Omore, Karin F Perez Garcia, Farah Naz Qamar, Syed Qudrat-E-Khuda, Sonia Qureshi, Md Nazmul Hasan Rajib, Wagner Valentino Shapiama Lopez, Shazia Sultana, Desiree Witte, Mohammad Tahir Yousafzai, Alex O Awuor, Nigel A Cunliffe, M Jahangir Hossain, Maribel Paredes Olortegui, Milagritos D Tapia, K Zaman, Arianna Rubin Means

**Affiliations:** Department of Global Health, University of Washington, Seattle, Washington, USA; Department of Pediatrics and Child Health, Aga Khan University, Karachi, Pakistan; School of Global and Public Health, Department of Health Systems and Policy, Kamuzu University of Health Sciences, Blantyre, Malawi; Infectious Diseases Division, International Centre for Diarrhoeal Disease Research,Bangladesh Dhaka, Bangladesh; Medical Research Council Unit The Gambia at the London School of Hygiene & Tropical Medicine, Fajara, The Gambia; Kenya Medical Research Institute, Center for Global Health Research (KEMRI-CGHR), Kisumu, Kenya; Asociación Benéfica PRISMA, Iquitos, Peru; Centre pour le Développement des Vaccins du Mali (CVD-Mali), Bamako, Mali; Department of Global Health, University of Washington, Seattle, Washington, USA; Medical Research Council Unit The Gambia at the London School of Hygiene & Tropical Medicine, Fajara, The Gambia; International Vaccine Access Center, Johns Hopkins Bloomberg School of Public Health, Baltimore, Maryland, USA; Medical Research Council Unit The Gambia at the London School of Hygiene & Tropical Medicine, Fajara, The Gambia; Asociación Benéfica PRISMA, Iquitos, Peru; Department of Global Health, University of Washington, Seattle, Washington, USA; Centre pour le Développement des Vaccins du Mali (CVD-Mali), Bamako, Mali; Medical Research Council Unit The Gambia at the London School of Hygiene & Tropical Medicine, Fajara, The Gambia; Centre pour le Développement des Vaccins du Mali (CVD-Mali), Bamako, Mali; Centre pour le Développement des Vaccins du Mali (CVD-Mali), Bamako, Mali; Infectious Diseases Division, International Centre for Diarrhoeal Disease Research,Bangladesh Dhaka, Bangladesh; Division of Infectious Diseases and International Health, School of Medicine, University of Virginia, Charlottesville, Virginia, USA; Center for Vaccine Development and Global Health, University of Maryland School of Medicine, Baltimore, Maryland, USA; Department of Pediatrics, University of Maryland School of Medicine, Baltimore, Maryland, USA; Department of Medicine, University of Maryland School of Medicine, Baltimore, Maryland, USA; Center for Vaccine Development and Global Health, University of Maryland School of Medicine, Baltimore, Maryland, USA; Malawi Liverpool Wellcome Programme, Blantyre, Malawi; Malawi Liverpool Wellcome Programme, Blantyre, Malawi; Kenya Medical Research Institute, Center for Global Health Research (KEMRI-CGHR), Kisumu, Kenya; Centre pour le Développement des Vaccins du Mali (CVD-Mali), Bamako, Mali; Kenya Medical Research Institute, Center for Global Health Research (KEMRI-CGHR), Kisumu, Kenya; Asociación Benéfica PRISMA, Iquitos, Peru; Department of Pediatrics and Child Health, Aga Khan University, Karachi, Pakistan; Infectious Diseases Division, International Centre for Diarrhoeal Disease Research,Bangladesh Dhaka, Bangladesh; Department of Pediatrics and Child Health, Aga Khan University, Karachi, Pakistan; Infectious Diseases Division, International Centre for Diarrhoeal Disease Research,Bangladesh Dhaka, Bangladesh; Asociación Benéfica PRISMA, Iquitos, Peru; Department of Pediatrics and Child Health, Aga Khan University, Karachi, Pakistan; Malawi Liverpool Wellcome Programme, Blantyre, Malawi; Institute of Infection, Veterinary and Ecological Sciences, University of Liverpool, Liverpool, UK; Department of Pediatrics and Child Health, Aga Khan University, Karachi, Pakistan; Kenya Medical Research Institute, Center for Global Health Research (KEMRI-CGHR), Kisumu, Kenya; Institute of Infection, Veterinary and Ecological Sciences, University of Liverpool, Liverpool, UK; Medical Research Council Unit The Gambia at the London School of Hygiene & Tropical Medicine, Fajara, The Gambia; Asociación Benéfica PRISMA, Iquitos, Peru; Center for Vaccine Development and Global Health, University of Maryland School of Medicine, Baltimore, Maryland, USA; Department of Pediatrics, University of Maryland School of Medicine, Baltimore, Maryland, USA; Department of Medicine, University of Maryland School of Medicine, Baltimore, Maryland, USA; Infectious Diseases Division, International Centre for Diarrhoeal Disease Research,Bangladesh Dhaka, Bangladesh; Department of Global Health, University of Washington, Seattle, Washington, USA

**Keywords:** cost, cost of illness, diarrhea, health economics, *Shigella*

## Abstract

**Background:**

Comparative costs of public health interventions provide valuable data for decision making. However, the availability of comprehensive and context-specific costs is often limited. The Enterics for Global Health (EFGH) *Shigella* surveillance study—a facility-based diarrhea surveillance study across 7 countries—aims to generate evidence on health system and household costs associated with medically attended *Shigella* diarrhea in children.

**Methods:**

EFGH working groups comprising representatives from each country (Bangladesh, Kenya, Malawi, Mali, Pakistan, Peru, and The Gambia) developed the study methods. Over a 24-month surveillance period, facility-based surveys will collect data on resource use for the medical treatment of an estimated 9800 children aged 6–35 months with diarrhea. Through these surveys, we will describe and quantify medical resources used in the treatment of diarrhea (eg, medication, supplies, and provider salaries), nonmedical resources (eg, travel costs to the facility), and the amount of caregiver time lost from work to care for their sick child. To assign costs to each identified resource, we will use a combination of caregiver interviews, national medical price lists, and databases from the World Health Organization and the International Labor Organization. Our primary outcome will be the estimated cost per inpatient and outpatient episode of medically attended *Shigella* diarrhea treatment across countries, levels of care, and illness severity. We will conduct sensitivity and scenario analysis to determine how unit costs vary across scenarios.

**Conclusions:**

Results from this study will contribute to the existing body of literature on diarrhea costing and inform future policy decisions related to investments in preventive strategies for *Shigella*.

## Teaser

The Enterics for Global Health (EFGH) *Shigella* surveillance study will estimate the cost per episode of medically attended *Shigella* diarrhea across 7 countries to inform policy decisions about potential investments in preventive strategies.

*Shigella* spp. are gram-negative bacteria that cause bacillary dysentery or shigellosis. Shigellosis is associated with linear growth faltering in children and is responsible for an estimated 60 000 deaths of children <5 years old each year [1]. The majority of cases occur in low-and-middle-income countries (LMICs), where access to care, relevant diagnostics, and treatment facilities are limited. An estimated one-third of children in low-resource settings experience ≥1 episode of *Shigella-*attributable diarrhea during their first 2 years of life [2]. Global estimates suggest that >2.1 million cases of moderate-to-severe stunting in LMICs are attributable to shigellosis annually [3].

The average cost for pediatric diarrhea management in outpatient and inpatient facilities in LMICs is approximately $40 (USD) and $160 per episode, respectively, with costs varying widely across studies due to different methodological approaches to data collection and analysis [4]. Variations in estimated costs are also observed across primary, secondary, and tertiary level healthcare settings owing to differences in disease severity presenting at each level. Estimated costs are $3.70, $33.79, and $31.86 per episode for outpatient settings, respectively and $30.70, $144.54, and $154.89 per episode for inpatient settings, respectively (in 2015 USD). However, there is minimal evidence for costs associated with *Shigella-*attributed diarrhea specifically. A study of costs associated with shigellosis patients of all ages was conducted in 3 Thai public hospitals. Researchers used a microcosting approach from the hospital (health system) perspective to capture direct medical costs of laboratory tests, medications, and health service delivery (nursing care, hotel components of inpatient services, etc) and used stepwise multiple regressions to generate a cost function. They estimated direct treatment costs of culture confirmed *Shigella* diarrhea to be $8.65 (in 2006 USD) across all patients, with outpatient visits estimated at $3.51 and inpatient admissions at $63.25 [5].

Costs associated with diarrheal illness are borne not only by health facilities and health systems but also by households (ie, caregivers and other family members). Pediatric diarrhea, including *Shigella-*attributed diarrhea, requires families to forego income-generating activities and travel to a healthcare facility with an ill child, receive an appropriate diagnosis, and access treatment—namely, rehydration therapy and antibiotics—from a health facility or private pharmacy. This cascade of activities to access treatment can be impoverishing, particularly in severe cases [6, 7].

The Global Enteric Multicenter Study (GEMS), a prospective matched case-control study in 7 country sites across Asia and Africa, evaluated pathogen-specific costs borne by households with a child aged 0–59 months experiencing diarrhea [8]. Household costs included out-of-pocket expenses for the child's medical care, such as transportation to healthcare providers, consultations, drugs, diagnostics, food, and other related costs. Households enrolled in GEMS incurred costs due to *Shigella*-attributable diarrhea averaging $8.44 for outpatient visits and $17.94 for inpatient visits (in 2012 USD). There were no significant differences in household costs between pathogens within a country.

There are considerable gaps in the costing evidence base for pediatric diarrhea generally and *Shigella-*attributed diarrhea specifically. The dearth of evidence from studies using similar methodological approaches limits generalizability [4]. Additional data points are needed from heterogenous settings to understand drivers of costs from both healthcare provider and household perspectives, including care-seeking history, disease severity, patient age or other vulnerability indicators, and treatments prescribed. This evidence is necessary to understand the potential value proposition of a future *Shigella* vaccine and the potential costs that could be averted for health systems and households should a vaccine or other innovative interventions become available in high-burden settings. The purpose of the current analysis is to provide a comprehensive cost estimate of shigellosis across multiple diverse settings to inform future policy decisions about potential investments in preventive strategies.

The Enterics for Global Health (EFGH) *Shigella* surveillance study will use cross-sectional and longitudinal data collection to establish incidence and consequences of *Shigella* medically-attended diarrhea (MAD) within 7 country sites in Africa, Asia, and Latin America. The overarching objective of this embedded costing study is to estimate costs associated with *Shigella* diarrhea incurred by households and healthcare systems.

## METHODS

### Study Setting

The EFGH study will be conducted over a 24-month period and will enroll an estimated 1400 children between 6–35 months old being treated for diarrhea at health facilities in sites located in 7 countries, including Peru, Pakistan, Bangladesh, Mali, Malawi, Kenya, and The Gambia (approximately 9800 children total). The study will collect data from 29 health facilities (5 in Bangladesh, 6 in Kenya, 1 in Malawi, 4 in Mali, 6 in Pakistan, 5 in Peru, and 2 in The Gambia). Enrollment sites include public and nonprofit facilities, representing primary, secondary, and tertiary levels of healthcare. More information about healthcare systems in each country involved in EFGH are described elsewhere [9–15].

### Perspectives

We will present costs of *Shigella*-attributable MAD from multiple perspectives, including: (1) the perspective of families of children experiencing diarrhea (“households”), (2) the perspective of health facilities engaged in the EFGH study (“health system”), and (3) a societal perspective that includes both household and health system costs.

### Time Horizon

The EFGH study will collect data over a 24-month surveillance period, which accounts for seasonal variability in costs [16]. We will not project costs beyond the study time period for the purposes of this analysis, however we may conduct future substudies using dynamic modeling or other methods for projecting costs within different vaccine coverage scenarios.

### Protocol Development

The EFGH team have developed standardized study tools—including a central protocol, standardized operating procedures, and data collection tools—using working groups comprised of subject matter experts from each site [16]. Representatives involved in an EFGH working group on diarrhea case surveillance provided input on costing methods and supported the development of survey questions. Each site identified a costing lead who will provide ongoing support for the costing aim throughout data collection and analysis.

### Data Collection

We will collect and categorize costs associated with MAD episodes as direct medical costs, direct nonmedical costs, and indirect costs. Direct medical costs include medications, diagnostics, personnel, and hospital bed-day costs borne both by the health system and households. These costs include all costs associated with assessment and treatment, irrespective as to whether the diagnostics or treatments were clinically indicated, as these represent costs that could be averted by a future vaccine that prevents a MAD episode. Direct nonmedical costs mainly entail travel costs borne by caregivers and family members. Indirect costs, sometimes called opportunity costs, are the value of time lost from productive work. We will estimate time lost from work for caregivers (eg, the primary family member caring for the child during their illness) of children with diarrhea.

To determine resource use, we will make use of the following EFGH data sources: (1) clinical treatment records, (2) data collected at the enrollment visit, on discharge from the health facility, and at follow-up visits 4 weeks and 3 months post-discharge, and (3) mortality interviews with families. To estimate values of resources used, we will use facility or national-level price lists, the WHO-CHOICE (CHOosing Interventions that are Cost-Effective) database, and existing EFGH data collection tools (Table 1) [17].

**Table 1.** Overview of Major Costs and Associated Data Sources

Type of Cost	Data Source
Direct medical costs	
Type of visit (inpatient vs outpatient)	Clinical treatment records
Time spent in care	Clinical treatment records
Health service delivery cost per outpatient visit	WHO-CHOICE
Health service delivery cost per day of inpatient visit	WHO-CHOICE
Drugs administered or prescribed at visit for treatment of diarrhea	Clinical treatment records
Diagnostics performed at visit to determine cause of diarrhea	Clinical treatment records
Cost per drug and diagnostic	National price lists, primary data collection from facilities
Estimate of caregiver fees associated with visit	Clinical treatment records, discharge survey, 4-wk follow-up survey
Direct nonmedical costs	
Cost of transportation to access health facility (eg, bus)	Enrollment survey
Costs associated with accommodation at facility for family (food, lodging, etc)	Discharge and 4-wk follow-up surveys
Indirect costs	
Estimate of caregiver lost time associated with caring for child during diarrhea episode	Discharge survey
Estimate of average wage rate in country	International Labor Organization
Pre- and post-visit care-seeking costs	
Costs of care seeking before and after a facility visit, by expense (medication, diagnostic tests, etc) and visit type (eg, visit to pharmacy shop, private clinic, or traditional healer)	Enrollment, discharge, and 4-wk follow-up surveys

#### Direct Medical Costs: Drugs and Diagnostics

Direct medical costs include costs incurred when diagnosing and treating children with MAD, in either outpatient or inpatient settings. Direct costs can be incurred by the health system and/or can be out-of-pocket costs incurred by households if drugs or medical items are needed but not provided by health facilities [18]. We will estimate the costs of drugs and any diagnostics used in the assessment and treatment of enrolled children, primarily from national price lists. Governments maintain price lists for medications used by public hospitals and facilities and update the lists periodically to reflect price changes. Because the prices are based on large volume government purchases, price lists typically approximate actual economic costs (unlike market prices of drugs purchased at private facilities, which may be overestimates of true costs due to profit margins) [19].

In the event that certain medications, supplies, or diagnostic tests are not included on national price lists, we will conduct health facility surveys to obtain data on the purchase prices of drugs and materials at each facility, as well as the prices charged by private laboratories. If both national price lists and reliable data on purchase prices are unavailable, then we will use standardized international price lists, which include a range of prices from international suppliers for common medications, to estimate the cost of a mediation or diagnostic test [19].

#### Direct Medical Costs: Health Service Delivery Costs

Health service delivery costs include the “hotel” component of hospital stays and operational costs for outpatient stays, excluding patient-specific diagnostic tests, medications, and medical supplies (described above). We will estimate health service delivery costs using EFGH data and WHO-CHOICE [17].

The WHO-CHOICE project developed standardized country-level estimates of service-delivery costs across diseases and treatments, presented as the cost per inpatient bed-day and the cost per outpatient visit. WHO-CHOICE inpatient bed-day and outpatient cost estimates include personnel, capital infrastructure and equipment, laboratory, maintenance of facility equipment and building amenities, food costs, and other operational costs [17]. WHO-CHOICE estimates both inpatient and outpatient care costs for different levels of the health system (eg, primary, secondary, and tertiary-level facilities). These estimates were originally generated via data from hospital-based costing studies in 49 countries for various years ranging from 1973 to 2000, amounting to 2173 country-years of observations [20]. Regression models were used to predict costs in countries for which primary data are not yet available. Models have been updated over time using new data and refined methods [17, 21]. The project includes primary data from Bangladesh, Kenya, Malawi, and Pakistan, Peru, and The Gambia, while costs from Mali are modeled estimates.

The Institute for Health Metrics and Evaluation (IHME) similarly developed cost-comparative estimates of services delivery costs [22]. On average, country-specific costs per outpatient visit from IHME are approximately 103% higher than estimates from WHO-CHOICE, and the costs per admission are 3% higher [22]. This is in part due to the fact that IHME costs include ancillary services such as diagnostics and drugs, whereas WHO-CHOICE estimates exclude these costs. Because EFGH is collecting detailed data on diagnostics and medication use, WHO-CHOICE is more closely aligned with health service delivery costs currently unaccounted for. WHO-CHOICE health service delivery cost estimates have also been used in multiple economic evaluations where primary data collection was not practicable, including treatment of dysentery across regions [4, 23].

The EFGH study will estimate inpatient bed-days and outpatient visits using WHO-CHOICE country-specific estimates. As WHO-CHOICE estimates are presented in 2010 international dollars, we will convert and inflate estimates to present-day US dollars [24]. To compute inpatient cost estimates, we will multiply WHO-CHOICE daily inpatient service delivery costs by the number of days a child was hospitalized (extracted from medical records). We will also compute outpatient cost estimates by multiplying the WHO-CHOICE standardized country rate by the number of outpatient visits observed.

#### Direct Medical Costs: Pre– and Post–Care Seeking Costs

At the time of enrollment, we will ask caregivers to report any prior care they sought for the diarrhea episode, including costs of visiting a pharmacy or drugs purchased before the visit. During follow-up, we will similarly ask caregivers about additional care they sought after their child's initial EFGH enrollment visit (Table 1). Cost estimates of care seeking before and after visits may be less precise than estimates for observed EFGH study visits owing to potential recall bias and less detailed reporting of resource use. Therefore, we will conduct sensitivity analyses using plausible ranges of health system costs and household expenditures during these periods.

#### Direct Nonmedical Costs

Direct nonmedical costs include payments borne by the patient's primary caregiver and other family members, such as transportation to the health facility and costs associated with food and accommodation for families while their child is treated at a facility [18]. These costs are not reimbursed by insurance and can exacerbate the financial burden to households [7]. In EFGH, we will estimate these costs via caregiver reporting during enrollment, discharge, and follow-up (Table 1). Together with direct medical costs borne by households, these costs constitute household out-of-pocket expenses.

#### Indirect Costs

Indirect costs describe productivity losses due to morbidity. We will estimate indirect costs from the household perspective, specified as the value of the time lost by caregivers from income-generating activities during the child's illness (eg, opportunity costs) [18]. Costing studies have commonly overlooked indirect costs, but wages that go unearned make up a substantial portion of the economic losses associated with diarrheal illness [4, 6]. In LMICs, indirect costs of medical treatment are estimated to be 2–3.6 times greater than direct costs [25].

We will use a human capital approach to estimating indirect costs, meaning we will estimate productivity losses using a caregiver's market value (eg, current estimated earnings) and their time lost from work during their child's episode of diarrhea [26]. Lost earnings will be measured as the sum of the total number of days of work lost due to their child's episode of diarrhea and associated care seeking, multiplied by the average local daily wage rate [26]. We will use EFGH surveys with caregivers during facility and follow-up visits to collect data about the total number of days of work lost. The average local daily/hourly wage rate will be estimated based on national labor force surveys uploaded to the International Labor Organization, which include all sectors of the economy and all categories of workers [27].

#### Costs Excluded From This Analysis

We will classify all resources related to treatment of MAD diarrhea in the EFGH study as either routine costs or research costs. Routine costs will include expenditures associated with clinical examinations performed during enrollment, anthropometric measurements taken at the time of enrollment, specimen collection, treatment and follow-up care. Research costs will be excluded from the analysis, as they do not reflect costs from the health system or household perspectives, including resources related to obtaining consent, conducting caregiver interviews, abstracting records, or nonindicated diagnostic tests. For example, we will exclude any laboratory tests conducted as part of the study that would not typically be carried out for a given patient. We will also exclude costs exclusively related to non-*Shigella* comorbid conditions, such as malaria treatment.

#### Shigella Attribution

All MAD costs will be stratified by confirmed *Shigella* status. *Shigella-*attributed MAD costs will include children with culture-confirmed *Shigella* and children with molecularly confirmed *Shigella* MAD. Additional details on EFGH methods for *Shigella*-attribution are described elsewhere [28, 29].

### Analysis

We will generate descriptive summary statistics (means, proportions and corresponding 95% confidence intervals) for sociodemographic characteristics of children presenting with MAD across sites by age and level of care. Details of these characteristics are described elsewhere [16].

We will calculate the average cost per *Shigella* diarrhea episode treated, referred to henceforth as the “unit cost.” Unit costs will be averaged across all enrolled patient visits (estimated enrollment between June 2022 and June 2024). Before averaging unit costs, we will conduct descriptive statistics to explore the distribution of costing inputs (eg, travel and antibiotic costs). We expect that costs will be right (positively) skewed, meaning that some patients incur substantially higher costs than the median, but not substantially lower costs [30]. We will use box plots to identify outlier costs incurred, defined as within 25%–75% of the cost distribution [31]. In sensitivity analyses, we will replace extreme values at the second and 98th percentiles by applying the cost of the second percentile to observed costs less than that value and applying costs of the 98th percentile to costs above that value, a process known as *winsorization* [31]. In a second sensitivity analysis, we will repeat these steps at the fifth and 95th percentiles to limit the effects of outlying values on estimates of central tendencies.

We will estimate the mean, standard deviation, median, minimum, and maximum unit cost values separately by site, from the health system perspective, the household perspective, and the societal perspective, which includes both health system and household-relevant costs [8, 32, 33]. We will also present costs separately by visit type, client characteristics, and disease outcome (recovery, rehospitalization, or death), as detailed in Table 2. For comparability across sites, we will present unit costs in 2024 USD. Costs incurred in the years before 2024 will first be inflated to 2024 values using gross domestic product price deflators from the World Bank [24, 34]. Midyear local currency values will then be used to convert local currency to equivalent 2024 USD values.

**Table 2.** Independent Variables to be Evaluated as Cost Drivers

Variable	Categories
Site	Bangladesh
	The Gambia
	Kenya
	Malawi
	Mali
	Pakistan
	Peru
Perspective	Household (out-of-pocket and indirect costs)
	Health system (health system–funded costs of treatment)
	Societal (includes both household and health system costs)
Visit type	Inpatient
	Outpatient
Level of care	Primary
	Secondary
	Tertiary
Disease outcome	Recovery
	Rehospitalization
	Death
Patient characteristics	Age
	Sex
	Diarrhea severity (mild, moderate, or severe illness)

We will explore variability in unit costs and drivers of costs through various methods. Given the anticipated skewed nature of the outcome of interest (unit costs), we will use multivariate generalized linear models to evaluate the effects of key variables on unit cost. Potential independent variables to be included in the model are country, level of facility, type of visit (inpatient vs outpatient), and illness severity.

In addition to unit costs of treatment, we will estimate the total economic burden of *Shigella* MAD in each EFGH site catchment area. We will calculate the economic burden as the product of the incidence of *Shigella* in the catchment area and mean societal unit costs, in each setting. Methods for estimating *Shigella* incidence are described elsewhere [16]. We will use these data and population enumeration data to generate an inflation factor necessary for estimating the total estimated costs of diarrheal care seeking and treatment in EFGH catchment areas. Additional analysis methods are detailed in the study's statistical analysis plan [35].

#### Sensitivity Analysis

We will conduct 1-way and 2-way sensitivity analyses to explore how altering key inputs affects overall costs. Inputs displaying high variability across visits (eg, caregiver-reported travel costs) and variables with high uncertainty (eg, caregiver wages) will be selected for sensitivity analyses. Specifically, we will explore how varying key costing inputs by 10% influence overall unit costs, and present unit cost variations in a tornado diagram. We will also explore costing inputs of policy relevance. For example, direct medical costs incurred when strictly following different clinical treatment guidelines may be modeled and compared with observed costs of providing care.

## DISCUSSION

Costs associated with MAD are considerable to health facilities, particularly where diagnostics are expensive and treatments require either hospitalization or referral to another facility [36, 37]. Diarrhea can also result in catastrophic or poverty-inducing expenditures for households, particularly in settings where families are responsible for bearing some or all treatment costs [6, 7]. Yet, detailed costing estimates of *Shigella* diarrhea are sparse and inconsistently reported in the literature, and no indirect costs have been estimated. This costing protocol aims to comprehensively estimate and describe the costs associated with *Shigella* management from the perspective of health facilities and families, identifying significant drivers of costs across settings and disease profiles.

A strength of this study is the opportunity to use consistent methods to collect and compare standardized costs across heterogenous settings, including diverse geographies, rural/urban facilities, and public/nonprofit settings. In addition, the study provides the opportunity to compare and triangulate across multiple data sources to derive and validate costs. In this study, comparing costs between data sources, such as a household survey and facility records, provides an opportunity to identify costs with highest uncertainty to inform sensitivity analysis and potentially address challenges in quality of self-reported data, such as recall bias.

The proposed study does have several limitations. First, we may observe higher resource use than would be observed outside of research settings, but we will also generate a more complete picture of the potential total costs of *Shigella*-associated management. Furthermore, costs generated in EFGH sites may not be nationally representative of diarrhea treatment costs, as these sites are disproportionately based in low-income areas and are limited to public sector and nonprofit facilities. While using national price lists to estimate drug costs reflects costs of drugs in EFGH study facilities, the market value of drugs—and therefore the cost of drugs purchased in private facilities or pharmacies—may be higher than national price estimates.

In addition, we rely on WHO-CHOICE to estimate direct health service delivery costs. While cost estimates would be most valid if new primary data were collected to estimate facility-specific bed day costs, it is logistically challenging to conduct detailed reviews of health facility budgets, client visit statistics, and time and motion studies in each setting. WHO-CHOICE, therefore, emerges as an alternative source of health service delivery costs and allows for impartial comparison across EFGH sites. Finally, this study is limited to estimating short-term costs related to *Shigella* MAD, and it will not estimate long-term costs related to diarrhea, such as potential long-term opportunity costs associated with growth faltering and other sequelae (eg, cognitive impairment and reduced income generation) [3, 38, 39].

While there are a number of evidence-based interventions available to treat *Shigella* diarrhea in low-income settings, implementation is inconsistent, in part owing to costs associated with care seeking and treatment resource availability [40, 41]. With antimicrobial resistance growing at an alarming pace, the development of a vaccine could be the most cost-effective strategy to reduce the impact of disease and associated long-term consequences [42, 43]. This study provides an important opportunity to understand resource use and identify the potential value proposition of a *Shigella* vaccine. Likewise, this study may provide evidence needed to conduct cost-effectiveness analyses that can guide the development of nonvaccine interventions (eg, WASH) to improve MAD or *Shigella* management while a vaccine is under development.
